# Information and Communication Technology Use and Economic Growth

**DOI:** 10.1371/journal.pone.0048903

**Published:** 2012-11-12

**Authors:** Maryam Farhadi, Rahmah Ismail, Masood Fooladi

**Affiliations:** 1 Department of Accounting, Mobarakeh Branch, Islamic Azad University, Isfahan, Iran; 2 School of Economics, Faculty of Economics and Management, Universiti Kebangsaan Malaysia, UKM Bangi, Selangor, Malaysia; Cinvestav-Merida, Mexico

## Abstract

In recent years, progress in information and communication technology (ICT) has caused many structural changes such as reorganizing of economics, globalization, and trade extension, which leads to capital flows and enhancing information availability. Moreover, ICT plays a significant role in development of each economic sector, especially during liberalization process. Growth economists predict that economic growth is driven by investments in ICT. However, empirical studies on this issue have produced mixed results, regarding to different research methodology and geographical configuration of the study. This paper examines the impact of Information and Communication Technology (ICT) use on economic growth using the Generalized Method of Moments (GMM) estimator within the framework of a dynamic panel data approach and applies it to 159 countries over the period 2000 to 2009. The results indicate that there is a positive relationship between growth rate of real GDP per capita and ICT use index (as measured by the number of internet users, fixed broadband internet subscribers and the number of mobile subscription per 100 inhabitants). We also find that the effect of ICT use on economic growth is higher in high income group rather than other groups. This implies that if these countries seek to enhance their economic growth, they need to implement specific policies that facilitate ICT use.

## Introduction

**Figure 1:**
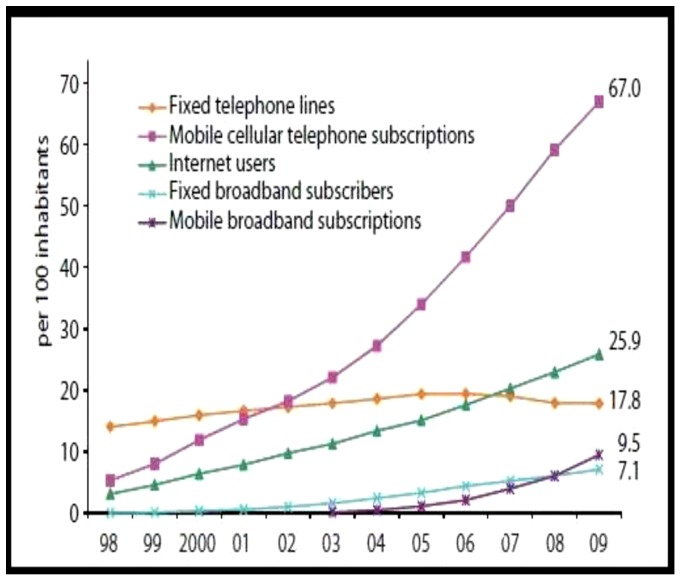
Global ICT developments, 1998–2009. Source: ITU World Telecommunication/ICT Indicators database.

At the present time, ICT has become a serious part of economy. Almost all firms and consumers use computers and Internet connection for economic purposes, such as providing consumers with a more diversified and customized products, improving product quality, and selling goods and services. Evidently, the extension of ICT and its influences on economic growth in both developed and developing countries has increased very fast during the last two decades. However, country data on computer, cell phone, and Internet users illustrate different ICT diffusion rates across countries and regions, ICT use indicators illustrate an increasing trend, despite the recent world economic crisis (Figure 1). For example, the steady growth of the number of mobile cellular subscriptions is noticeable, reaching 67 per 100 inhabitants by the end of 2009 globally. This confirms that consumers are willing to continue spending part of their disposable income on mobile services - even at times of financial constraints [1].

In addition, the resurgence of productivity during the late 1990s and the early 2000s is a topic that has attracted many growth economists. ICT is the current symbol of the technological revolution and is known as the key factor driving economic growth in the industrial society [2]. For measuring the contribution of ICT to economic growth, the most important issue is regarding to the specification of ICT.

ICT defines as a concept that include computers and other information equipment as well as computer software, that covers computers, peripheral equipment and other information-related office equipment (photocopiers, cash registers, calculators), communications equipment, and instruments [3], [4].

In fact, ICT is the combination of electronics, telecommunications, software, networks, and decentralized computer work stations, and the integration of information media [5], all of which impact firms, industries, and the economy as a whole. ICT is comprised of a variety of “communication equipment” which includes radio, TV, and communication equipment and software. Therefore, ICT investment includes “investments in both computer and telecommunications, as well as related hardware, software and services” [6].

In this article, we would like to examine the relationship between ICT use and growth rate of GDP per capita in 159 countries. Although many researchers have provided empirical evidences for the correlation between ICT investment and economic growth, study on the impact of ICT use on economic growth is still an unexplored area. Therefore, this article would fill the literature gap on the effect of ICT use by applying the panel of 159 countries over the period 2000–2009.

The organization of the paper is as follows: The next section is a review of relevant studies on the impact ICT on Economic growth. Afterward methodology and data will be presented which follows by the empirical findings and discussion. The last section concludes the article with a few issues on policy implications.

## Literature Review

The effect of ICT on economic growth has been analyzed by many authors in last decades. Most of the evidences in this area confirm that the positive effect of ICT on economic growth is not apparent before mid-1990s. Oliner and Sichel [7] use ICT capital components such as computer hardware, software and telecommunication equipment along with capital and labor as inputs and empirically verify a very high ICT contrition to economic growth in the late 1990s, but they find no evidence of a positive relationship before the mid-1990s. In 2000, Jorgenson and Stiroh [20] show that the contribution of IT in economic growth of the United States is because of the substitution of computers, related equipment and services, not due to technological change.

Moreover, other studies explain the significant effect of ICT on economic growth such as Brynjolfsson and Yang [8], Motohashi [9] and Kraemer and Dedrick [10]. Most of these studies have been reviewed by Pohjola [11]. Jalava and Pohjola [2] indicate that ICT use and production quality are the most important factors in US economic growth in the 1990s. In addition, they provide evidence that ICT boosts growth in Finland from 0.3% to 0.7% between the early and late 1990s.

In Europe, Schreyer [12] explores the impact of ICT capital and indicates that the contribution of ICT to economic growth of four European countries, the United States, Canada, and Japan during 1990–1996 is about 0.17–0.29%. Daveri [13] expands Schreyer's [12] study to 13 European and five others and shows a much higher contribution of ICT for each country. Both of them conclude that large European countries are far behind the US in this area. Applying a broad data set, Van Ark et al. [14] also confirm that the gains from ICT capital are higher in the US than in Europe.

Despite the numerous studies, the evidence of ICT contribution to economic growth in developing countries is still scarce. For instance, Dewan and Kraemer [15] estimate the effect of IT investment on output growth for the panel data of 36 countries over the period 1985–1993, and discuss the contrasting policy implications for IT capital investment by developed and developing economies. They reveal that return from IT capital investment is positive and significant for the developed countries in the sample but not statistically significant for the developing ones. This study attributes this gap to the low level of IT investment as well as lack of complementary assets in developing countries. They explain that complementary investments in infrastructure, human capital, and knowledge-based structures are prerequisite for IT investments to be productive which are mostly available in developed countries rather than developing ones.

Moreover, Lee et al. [16] indicate the significant impact of ICT on economic growth of many developed and Newly Industrialized Economies (NIEs), but not in developing countries. In line with this result, Edquist [17] conclude that the vague impact of ICT on economic growth in developing countries may account for the late introduction of ICT in these countries; for example, Internet service was not available in most developing countries until the late 1990s.

In contrast with the above discussion, Antonelli [18] suggests that developing countries may gain more benefit from ICT than developed countries since switching from the predominant technology to a new “ICT-oriented paradigm” enforce significant costs to developed countries. It can effectively lock developed countries into those paradigms while simultaneously, important opportunities open up for less-industrialized countries to catch up and even “leapfrog” beyond the industrialized countries because they have relatively lower switching costs [19]. In this point of view developing countries may have an advantage over advanced countries with respect to ICT diffusion.

Generally, we can divide the empirical evidence of the impact of ICT on economic growth to two categories based on the methodology used in these literatures. The first is studies employing the growth accounting technique, which weights growth in inputs by their share in the value of output and express the contribution of ICT to economic growth in percentage point. These studies comprise Jorgenson and Stiroh [20], Oliner and Sichel [7] and Jorgenson [21], for the United States; Jalava and Pohjola [11] for Finland; Oulton [22] for the United Kingdom; Colecchia and Schreyer [23], Daveri [24], Van Ark et al. [14] and Timmer et al. [25] for Europe; Jorgenson [26] for the group of seven (G7) countries; Jorgenson and Motohashi [27] for Japan; and Jorgenson and Vu [28] for 110 countries.

It should be noted that all the above evidences are at the national level whereas there are some other studies at the firm or industry level. For instance, O'Mahony and Vecchi [29], applying heterogeneous dynamic panels method with a unique dataset covering the entire non-agricultural market economy at the industry level for the US and UK from 1976 to 2000, find a positive and significant effect of ICT on economic growth and excess returns to ICT compared with non-ICT assets.

The second category consists of researches that use cross country regression techniques to investigate the impact of ICT on economic growth. Madden and Savage [30], using the sample of 27 Central and Eastern European countries, show a positive and significant impact of telecommunication investment on economic growth during the period 1990–1995. Roller and Waverman [31] also confirm a causal relationship among telecommunication investment and economic growth for 21 OECD countries over the period 1970 to 1990. Jacobsen [32] and Waverman et al. [33] in a similar study indicate a positive impact of mobile phones on economic growth. Another study conducted by Koutroumpis [34] for 22 OECD countries during 2002 to 2007, shows that there is a positive casual link among broadband infrastructure as a driving factor of ICT and economic growth, especially in the presence of critical infrastructure mass. Applying panel data of 29 countries, Seo et al. [35] investigate the bidirectional relationship between ICT investment and economic growth. They only verify the positive impact of ICT on economic growth in the 1990s. The positive and significant effect of mobile telecommunications diffusion on both economic growth and productivity growth has proven by Gruber and Koutroumpis [36] for 192 countries over the period 1990–2007.

Although ICT is well known as a driving engine of economic growth, there are few evidences that show the negative effect of ICT on economic growth. For example, Kiley [37], applying the traditional growth accounting framework in the US, explains the negative contribution of computers to economic growth due to adjustment costs. He indicates that the introduction of a new investment good like computers can impose large adjustment costs to the economy and decrease economic growth. Moreover, Pohjola [2] finds no significant relationship between ICT investment and economic growth for the sample of 43 countries over the period of 1985–1999. In another research, Jacobsen [32] reveals no significant positive impact of computer penetration on the economic growth of 84 countries during 1990–1999, although he confirms the positive link among mobile phone and growth.

However, the empirical results of the previous studies are somewhat fragile and depend on data period specifications and econometric techniques, the dominant impact of ICT as a production input on economic growth and productivity is positive [38], [39], [24], [40], [41], [42].

Evidently, most of the literatures in the field of ICT effect on economic growth and productivity, concentrate on the ICT investment as a whole and evidence on the impact of ICT use on economic growth and productivity is scarce. Only a few studies investigate the effect of ICT use on economic performance applying different proxies such as telephone penetration estimated by number of telephones per 100 persons [43] and teledensity defined as the number of fixed-line and mobile phone subscribers per 100 persons [44], [45]. No study to date has used ICT use index presented by ITU [46] to evaluate the impact of ICT use on economic growth.

Therefore, the main hypothesis of this paper is that the effects of ICT use (as measured by the number of internet users, fixed broadband internet subscribers and the number of mobile subscription per 100 inhabitants) on economic growth is positive and significant. We present results based on the Generalized Method of Moments (GMM) estimator. Combining data for the 159 countries, we find that ICT use has a positive impact on output growth.

## Methodology and Data

### Conceptual form

This study uses a dynamic panel data model [47] to investigate the impact of ICT use on economic growth. The model is shown as follows:
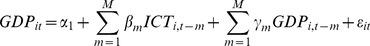
(1)where 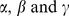 are the parameters to be estimated, GDP and ICT refer to natural logarithm of real GDP per capita and natural logarithm of ICT use indicator, respectively, and *m* indicate the level of lags for these two variables. *i* and *t* represent the countries in the sample, and the time periods. 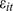 is the composite error term which is consists of 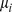 the unobserved country-specific effect, and 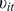 the idiosyncratic shocks (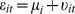).

In the above equation, the fixed effects (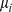), such as regional and demographics which are also called time-invariant country characteristics, might be correlated with the explanatory variables which violates the assumptions underlying the classical linear regression model [48]. Moreover with regards to dynamics, the presence of lagged dependent variables 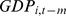 will increase the autocorrelation. In other words, 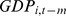
are correlated with the fixed effect in the error term and results in the “dynamic panel bias” [49]. First differencing can solve this problem by removing such fixed effects, as follows:
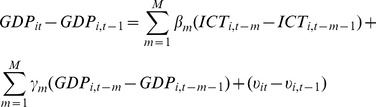
(2)There are still following econometric problems in the estimation of equation (2) which should be considered:

1- There is correlation between the new error term (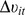) and the differenced lagged-dependent variable (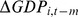).
2- Since our data set consists of 159 countries for 10 years; the dynamic pattern of the data cannot be ignored. Moreover, based on the dynamic nature of ICT, ICT use index shows wide variation. In this case the assumptions of stationarity of all the variables included in the regression and homogeneity of cross-country coefficients are violated.
3- This study encounters the endogeneity problem which caused by the measurement error of the ICT use index, which can produce biased estimated coefficients.

In this case, the simple ordinary least squares (OLS) approach can produce highly misleading results [50], [51]. Therefore, the empirical analysis for the estimation of equation (2) should employ a methodology that accounts for heterogeneous dynamic panels [52]. To overcome these issues, economists recommend the use of instrumental variables, and more recently panel data techniques such as Pooled Mean Group (PMG), discussed in Pesaran et al. [52] and Generalized Method of Moments (GMM) procedure of Arellano and Bond [53] introduced to address these problems more efficiently. However, when the number of cross-section observations is quite large and the time-series dimension is relatively small, as is the case in this paper, the GMM estimator can produce more consistent estimates [52]. Shortly, GMM estimator is useful for panel data with relatively small time dimension, as compared to the number of cross sections [54].

As a result of the above discussion, the Arellano-Bond [53] GMM method first proposed by Holtz-Eakin et al. [55] seems to be appropriate for the estimation of the equation (2), which addresses the problem of autocorrelation of the residuals and deals with the fact that some of the explanatory variables are endogenous. In this method lags of the dependent and independent variables are used as instruments. In this study, we consider lags up to four years and the dynamic panel data model is then applied to the complete panel dataset.

### Data

GDP per capita in US$ at constant 2005 prices, using the purchasing power parity (PPP) exchange rates has directly obtained from World Development Indicators [56]. This study calculates the ICT use index applying the Principal Component Analysis (PCA), following the two reports -measuring the information society in 2009 and 2010- presented by International Telecommunication Union (ITU). The ICT use index includes three indicators, Internet user penetration, fixed broadband penetration, and mobile broadband penetration and captures the level of ICT use in more than 150 countries worldwide. The ICT use indicators which are used to construct the index are all collected from ITU database. For more detail and background to the creation of ICT use index, one can refer to the ITU reports. The calculated ICT Use index based on ITU [46] formula which is one of the contributions of this research is presented in Table S1.

## Findings and Discussion

Our estimated results based on the GMM -dynamic panel data- are summarized in Table 1. Broadly, the results confirm the expected relationship between the real GDP per capita and ICT use index. As table 1 shows, the signs of all variables are consistent with theory predictions. The coefficient of ICT use index is positive and statistically meaningful at 5% significance level. It means that the more a country use ICT, the greater is its economic growth. The coefficient of ICT use index is equal to 0.17, indicates that if a country improves the ICT use index by one percent, the economic growth will increase 0.17%. Additionally, the coefficient of the first lagged ICT use index is equal to 0.09 which is also significant at 1% significance level. It means that one percent change in ICT use index of the previous year can increase the growth rate of GDP per capita by 0.09%. The statistics presented by the ITU and other international organizations indicate an increasing trend of ICT use indicators in most of these countries, it means that these countries recognized the important effect of ICT on their economic growth. They also verify the hypothesis of this paper that ICT use has a significant growth generating effect. The signs of the first lagged of ICT use index and GDP per capita coefficient are positive and highly significant that implies the positive effect of these variables on economic growth. Moreover the second, third and forth lagged of ICT and GDP are negative and mostly insignificant.

**Table 1:** Estimation Results using GMM Estimator.

Variable	Coefficient	Std. Err.
ICT	0.17	0.08**
ICT(−1)	0.09	0.03***
ICT(−2)	−0.08	0.07
ICT(−3)	0.01	0.05
ICT(−4)	0.01	0.03
GDP(−1)	1.93	0.39***
GDP(−2)	−1.19	0.38***
GDP(−3)	−0.07	0.14
GDP(−4)	0.04	0.11
Wald chi2(9)	31.99***	
Number of Obs.	790	
Number of groups	159	
Number of Instruments	17	
Arellano-Bond test for AR(1) in first differences: z = −4.10 Pr>z = 0.000		
Arellano-Bond test for AR(2) in first differences: z = 0.59 Pr>z = 0.555		
Hansen test of overid. restrictions: chi2(8) = 12.49 Prob>chi2 = 0.130		

***, ** and * denote statistically significant at 1%, 5% and 10%, respectively.

The dependent variable is the first-difference of the Ln(GDP) per capita and all variables are in Logarithm form.

GDP (-t) and ICT (-t), t = 1, 2, 3, 4 are lagged variables of GDP and ICT use index respectively.

In the context of GMM, the over-identifying restrictions may be tested via both the Sargan and Hansen test. The Sargan/Hansen test has a null hypothesis of “the instruments as a group are exogenous”. Therefore, the higher the p-value of the Sargan/Hansen statistic is the better [54]. In comparison the Hansen J statistic is more robust than Sargan. For example, Sargan is not distributed as chi-squared under heteroskedasticity and Hansen is, and if this problem is present then it could cause Sargan to incorrectly reject the null.

Consequently, this study employs the J-statistic of Hansen [57] which is distributed as χ2 with degrees of freedom equal to the number of over-identifying restrictions (L – K). L is the number of instrumental variables and K is the number of explanatory variables. A rejection of the null hypothesis shows that the instruments are not properly chosen. This may be either because they are not truly exogenous, or because they are being incorrectly excluded from the regression [58]. Based on the results in table 1, the Hansen J-statistic fails to reject the null hypothesis of correlation between residuals and instrumental variables. Therefore, the credibility of the results for interpretation is verified and the results can be interpreted in a high level of confidence.

Evidently, the full disturbance 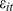 is presumed autocorrelated because it contains 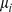, the fixed effects, and the GMM estimator is designed to deal with this problem. In order to test for autocorrelation aside from the fixed effects, we have applied the Arellano-Bond test to the residuals in differences. Since 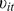 is mathematically related to 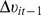 via the shared 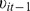 term, first-order serial correlation is expected in differences and evidence of it is uninformative. Therefore, to test the first-order serial correlation in levels, we should check the second-order correlation in differences. In general, we check for serial correlation of order *l* in levels by looking for correlation of order *l*+1 in differences.

The Arellano – Bond test for autocorrelation has a null hypothesis of no autocorrelation and based on the above discussion is applied to the differenced residuals. The test for AR(1) processes in first differences rejects the null hypothesis which is expected. The test for AR(2) in first differences is more important, because it will detect autocorrelation in levels. As indicated in table 1, we can reject the null hypothesis of the first-order serial correlation in levels or the second-order correlation in differences.

For further analysis of the impact of ICT use on economic growth, we categorized the sample of 159 countries into four different groups based on the per-capita income according to the classification of ITU [46]. The estimation results based on the GMM method for each group are summarizes in Table 2. Based on the table, in all the groups ICT use index has a positive and significant effect on economic growth which is in line with this paper hypothesis. Moreover, the ICT coefficient for the high income group is 0.11, which is the highest among the four income groups while, this coefficient for the low income group is just 0.02. It means that one percent change in ICT use index can increase the GDP per capita of a high income country 5.5 times more than a low income country.

**Table 2:** Estimation Results using GMM Estimator based on different income levels.

Variable	Coefficient			
Income level	High	Upper middle	Lower middle	Low income
ICT	0.11 (0.06)***	0.09 (0.04)**	0.06 (0.02)***	0.02 (0.01)*
ICT(−1)	0.02 (0.04)	0.08 (0.05)*	0.05 (0.02)***	0.005 (0.07)
ICT(−2)	0.02 (0.02)	−0.05 (0.02)***	−0.02 (0.02)	−0.001 (0.01)
ICT(−3)	−0.06 (0.02)***	0.05 (0.01)***	0.01 (0.02)	0.00 (0.006)
ICT(−4)	0.02 (0.02)	−0.03 (0.01)***	−0.01 (0.01)	−0.002 (0.004)
GDP(−1)	0.72 (0.088)***	0.56 (0.19)***	0.83 (0.05)***	0.33 (0.08)***
GDP(−2)	−0.18 (0.09)**	−0.38 (0.21)**	0.32 (0.07)***	0.11 (0.09)
GDP(−3)	0.26 (0.09)***	−0.09 (0.11)	−0.07 (0.07)	0.003 (0.08)
GDP(−4)	−0.37 (0.10)***	0.03 (0.07)	−0.08 (0.05)	0.04 (0.07)
Wald chi2(9)	643.54***	8929.47***	31412***	44406.14***
Obs.	245	216	258	181
Groups	49	36	43	31
Ins.	24	43	34	37
AR(1)	Z = −2.00***	Z = −1.74*	Z = 1.73*	Z = −2.47**
AR(2)	Z = 0.21	Z = −0.02	Z = −0.09	Z = 0.41
Hansen test	Chi2(15) = 17.70	Chi2(33) = 32.54	Chi2(25) = 22.48	Chi2(27) = 23.45

The dependent variable is the Ln(GDP) and all variables are in Logarithm form.

Figures in parentheses refer to standard errors.

***, ** and * denote statistically significant at 1%, 5% and 10%, respectively.

GDP (-t) and ICT (-t), t = 1, 2, 3, 4 are lagged variables of GDP and ICT respectively.

Table 2 also shows that the first lagged of ICT use index for all income groups is positive; however it is only significant in upper middle and lower middle income countries. These empirical results are consistent with the findings of Lam and Shiu [45].

**Figure 2:**
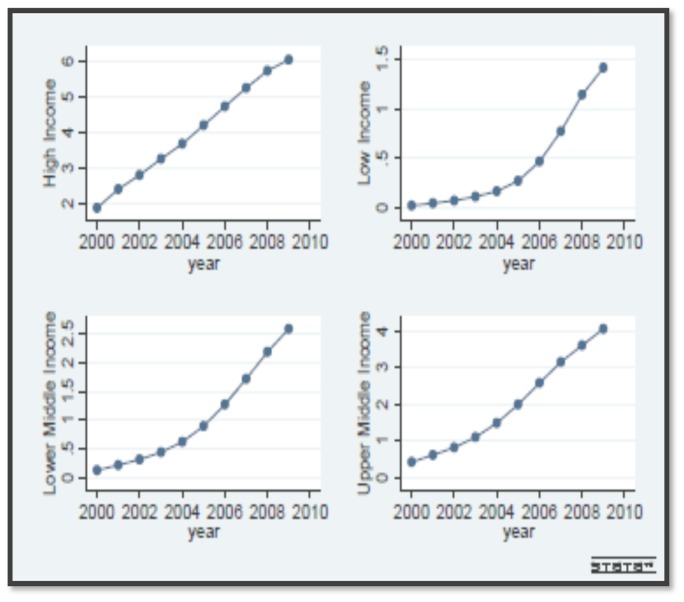
ICT use index, 2000–2009.

To shed more lights on the differences of countries regarding to ICT use index, following figures are presented. Figure 2 indicates the increasing trend of the ICT use index over the period 2000 to 2009 separately for each income group. As depicted in this figure, ICT use index has a nearly fixed growth in high income countries while other income groups have been experiencing an increasing growth of ICT use indicators.

**Figure 3:**
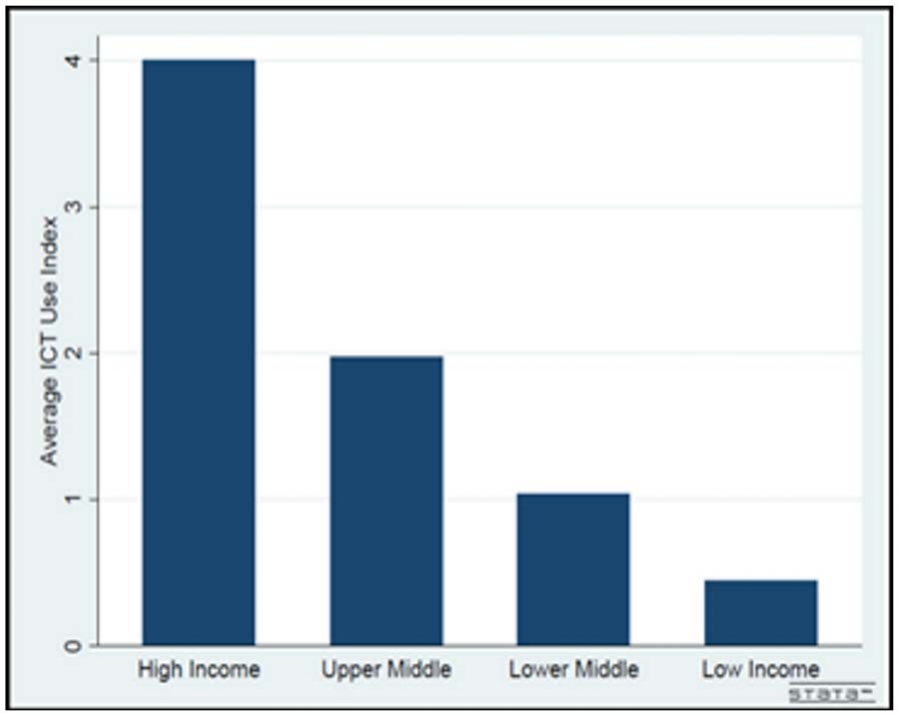
Average ICT use index by income groups.

Figure 3 also illustrates the different level of the average ICT use index in four income groups. As expected the highest value of the ICT use index is allocated to high income countries and the lowest value of this index is related to the group of low income countries. These findings can confirm the between groups differences in the estimated coefficients of ICT use index in table 2.

## Conclusions and Implications

This paper concentrated on exploring the effect of ICT use index on economic growth. The results show that ICT use has a significant effect on the economic growth of these countries. The coefficient measuring the effect of the ICT use on economic growth was positive, indicating that ICT affect economic growth of the 159 sample countries in a positive way. Furthermore, in high income counties ICT use index has the strongest effect on real GDP per capita among the others while this effect is the lowest in countries with low level of income. Moreover, the performance of the both higher middle and lower middle income groups in the effect of ICT use index is somewhat lagging. Therefore these countries can improve their overall GDP growth with policies aimed at increasing ICT use.

Consequently, ICT plays a vital role as a mean for economic growth. Therefore, it seems necessary for all countries to increase their ICT use index through increasing the number of internet users, fixed broadband internet subscribers and the number of mobile subscription per 100 inhabitants in order to boost economic growth. It is also essential for the governments to provide the society with information, up-to-date structures and educate people in order to use ICT efficiently. The major research limitation of this study was the failure to collect data for a longer time period. Therefore future research for a longer time span would shed more light in the assessment of the relationship between ICT use and economic growth.

## Supporting Information

Table S1 **ICT Use Index, 2000–2009.**
(XLSX)
